# A Semi-Supervised Approach for Improving Generalization in Non-Intrusive Load Monitoring

**DOI:** 10.3390/s23031444

**Published:** 2023-01-28

**Authors:** Dea Pujić, Nikola Tomašević, Marko Batić

**Affiliations:** Institute Mihajlo Pupin, University of Belgrade, Volgina 15, 11060 Belgrade, Serbia

**Keywords:** domain adversarial neural networks, generalization, non-intrusive load monitoring, semi-supervised learning

## Abstract

Non-intrusive load monitoring (NILM) considers different approaches for disaggregating energy consumption in residential, tertiary, and industrial buildings to enable smart grid services. The main feature of NILM is that it can break down the bulk electricity demand, as recorded by conventional smart meters, into the consumption of individual appliances without the need for additional meters or sensors. Furthermore, NILM can identify when an appliance is in use and estimate its real-time consumption based on its unique consumption patterns. However, NILM is based on machine learning methods and its performance is dependent on the quality of the training data for each appliance. Therefore, a common problem with NILM systems is that they may not generalize well to new environments where the appliances are unknown, which hinders their widespread adoption and more significant contributions to emerging smart grid services. The main goal of the presented research is to apply a domain adversarial neural network (DANN) approach to improve the generalization of NILM systems. The proposed semi-supervised algorithm utilizes both labeled and unlabeled data and was tested on data from publicly available REDD and UK-DALE datasets. The results show a 3% improvement in generalization performance on highly uncorrelated data, indicating the potential for real-world applications.

## 1. Introduction

As ecology, environmental protection, and energy efficiency gain more prominence, so does research into energy saving. It has been claimed that approximately 40% of the world’s electricity consumption can be attributed to residential and commercial buildings [1]. Recent research has focused on residential buildings and has suggested that energy consumption can be reduced by up to 12% just by giving feedback to residential customers on ways in which they spend their energy, pointing out which home appliances use energy at each point in time [2]. Therefore, to provide that necessary information, the most powerful approach, called intrusive load monitoring (ILM), proposes connecting smart meters, sensors for measuring consumed energy, to every appliance in the household/office/etc. This approach enables obtaining high-resolution and accurate energy information, which could be beneficial, especially in industrial applications, as suggested by [3]. However, in cases when it is not possible to apply ILM, for example, in the residential sector when it is cost-ineffective or unappealing for the end users to install smart meters on all appliances in the households, energy consumption disaggregation is required and, hence, the concept of non-intrusive (appliance) load monitoring (NI(A)LM) is defined. Namely, NILM attempts to collect the same information using only aggregated power measurement, or in other words, the main goal of NILM is in solving the energy disaggregation problem on the appliance level. However, the precision of NILM approaches significantly decreases when data distribution from the targeting domain differs from the source one, which could negatively influence NILM’s utilization in everyday life. Therefore, the domain adversarial neural network approach (DANN) is proposed in this paper to improve generalization performances to extend the real-world application of NILM approaches.

Hence, the main contributions of this paper are as follows:

• Proposition and implementation of DANN approach for the first time in the context of the NILM problem;
• Solving one of the crucial NILM problems of achieving better generalization by combining beneficial characteristics of both supervised and unsupervised learning approach, exploiting the full potential of accessible data;
• Benchmarking the system’s performance against the current state-of-the-art methodology using two real-world publicly available (and most used) datasets;
• Establishing an approach that makes NILM more applicable in everyday life, making it more convenient in practice than previously proposed solutions.

The remainder of this paper is organized as follows. Section 2 provides an extensive state-of-the-art review for non-intrusive load monitoring, while Section 3 elaborates on the problem definition, current difficulties, technical barriers, and the main problems of this paper. Details regarding the proposed approach are given in Section 4, i.e., the method, architecture, and training specificity, whilst in Section 5, data preprocessing methods, characteristics, and parameters are discussed along with the essential measurement data characteristics that can potentially influence the results and performances. Section 6 presents an overview of the training specification and description, followed by the results and performances presented in Section 7. Finally, Section 8 summarizes the paper, giving the overall view and conclusion of the obtained results and providing future work suggestions.

## 2. Related Works

The first field that will be covered by the state-of-the-art analysis is related to the hardware-based methods for individual consumption measurements—the ILM methods, reviewed in [4]. These are focused on the deployment of IoT sensors on household appliances to obtain crucial measurements, further enabling the development of various IoT-based applications. In [5], IoT architecture consisted of the appliance layer, perception layer, communication network layer, middleware layer, and application layer enables the development of the activity recognition system based on energy-related data. Moreover, in [6], the authors offered a rich data set, which combined outputs of ILM, or energy-related data, and other IoT measurements, such as indoor environmental conditions, HVAC operations, and outdoor weather conditions. This gives the possibility to exploit the data for occupancy predictions, behavior modeling, building simulation, energy forecasting, etc. Furthermore, in [7], control strategies for reducing energy consumption are based on energy consumption measurements by the Plug-Mate management system, showing the potential and effectiveness of ILM.

Even though it is clear from the previous related work summary that the application of ILM approaches are wide, there are, unfortunately, cases when the installation of a large number of sensors is not possible and, hence, ILM could not be established, which is why NILM modeling is attractive and common in the literature.

The first method dealing with the NILM problem was introduced in the late 20th century by Hart [8], whose idea was to detect appliance activations based on the magnitude of the difference in aggregate power. Even though this approach can be easily modified to suit multi-state devices, those which have more than one steady state, this approach is hardly practical, as the difference in magnitude is not equal for all types of the same device. For example, appliances from different manufacturers usually do not consume the same amount of power in the same state. Another huge problem in this approach involves the so-called continuous devices, where consumption varies through time, taking an infinite number of potential states, such as a refrigerator. Accordingly, numerous techniques were developed to deal with these problems; in the literature, they are separated into two groups, i.e., methods using data sampled with high and low sample rates [9]. By the term high sample rate, a range of frequencies from several kHz to several MHz is considered. These approaches are appealing as they can include frequency-based techniques and analyze transient characteristics in more depth. However, smart meters with such a high sampling rate are generally not available in households nowadays, making these approaches non-applicable in practice. On the other hand, low sampling rate methods, with sampling frequencies of 1 Hz and lower, are the main point of interest of researchers in the NILM field nowadays. Apart from the classification by sampling rate, due to different characteristics and the amount of publicly accessible data, the algorithms can be separated by their practical uses into ones developed for the residential sector and others for the commercial sector [10]. As the main focus of this paper lies within the residential sector, the commercial sector is not going to be discussed any further.

The first group of low sampling rate methods that are widespread in the literature involve techniques based on hidden Markov models (HMMs). A HMM is a Markov model with non-observable states. Alternately, the state is characterized by a probability distribution function that models the observation corresponding to that state [9]. HMMs are widely used because the state of the individual appliance is not directly observable, but can be obtained through aggregated power. In order to overcome computational complexity, which exponentially grows with the number of modeled devices, factorial hidden Markov models (FHMMs) were proposed in [11,12]. Moreover, to compensate for the inadequately modeled state occupancy duration, semi-hidden Markov models were presented in [13]. Additionally, to include the appliance correlation, conditional factorial hidden Markov models (CFHMM) were implemented and discussed in [14]. Moreover, [15] presented results using a combination of HMM and the improved Viterbi algorithm. Finally, in [16], the authors recently proposed FHMM based on adaptive density peak clustering, which reduces the dependence on prior information and is more applicable in real-world scenarios. Nevertheless, none of these methods learn appliance-specific patterns that significantly reduce the possibility of generalization. Furthermore, the detection of multi-state device activation is challenging for these algorithms as they do not adhere to the Markov assumption that the next state depends only on the current state, and not the previous ones.

Besides the different HMM approaches, other unsupervised methods are present in NILM literature, as well [17]. They are practically beneficial as they do not require individual device’s power consumption, which is likely to be inaccessible. On the contrary, they cannot achieve as high performances as the supervised ones, due to the lack of information. In [18], the authors presented a fully unsupervised approach based on clustering and histogram analysis using conditional random fields, whilst Dynamic Time Warping transformation and dynamic programming were employed for template signature matching in [19]. Moreover, in [20], the authors presented an unsupervised novel approach based on spiking deep NNs, which outperformed even some supervised ones. In [21], the hybrid approach was proposed, a combination of supervised and unsupervised methods, which is correlated with the work in this paper as our goal and one of the main contributions is improving generalization by using both labeled and unlabeled data. Nevertheless, the main concepts are entirely different as their approach is based on HMM, whilst the one in this paper is based on neural networks. Additionally, similarly to this paper, [22] focused on semi-supervised learning using both labeled and unlabeled data to improve the performances on the house of interest. However, their approach is focused on a semi-supervised one-nearest methodology, rather than convolutional neural networks, which were chosen as part of this work. Finally, [23] highlighted that there was a lack of semi-supervised approaches that exploited all accessible data.

Besides the HMM-based approaches, another probabilistic method that has been used in the NILM field is graph signal processing (GSP) as in [24,25,26], mostly all by the same authors. In [27], the authors highlighted potential problems of the model’s underperformance in real-world practice in the case when training data were lacking, proposing GSP with a novel learning algorithm as an appropriate solution for the NILM problem. Furthermore, in a recent study [28], GSP, enhanced by improving feature selection through extracting state transition sequence features, showed performance improvement on the publicly available data. Additionally, apart from these probabilistic methods, as reviewed in [29], many data-based techniques are proposed for solving the NILM problem, such as support vector machine (SVM), k-nearest neighbor (kNN), algorithms for matching the power signal with the built signature database, etc. Moreover, for feature detection, wavelet transformation [30] and the V-I trajectory approach [31,32] were proposed and employed for energy disaggregation, whilst in [33], the authors used the sum-to-k constraint to extract information from the bewildering combinations of different sources. Additionally, in [34], the Karhunen–Loève expansion (KLE) was used in combination with the spectral clustering-based method.

Nonetheless, neural networks (NNs), capable of solving tortuous problems, and making remarkable improvements in image classification and speech recognition, became some of the most frequently used state-of-the-art techniques due to the fact that they provide features that improve performances dramatically, as stated in [35,36]. Various papers explored different neural network architectures. One of the first was [37], where three different architectures were proposed based on convolutional neural networks (CNNs) and recurrent neural networks (RNNs). Their scores outperformed FHMM, proving that NNs are capable of extracting signatures that increase accuracy in detecting and estimating appliance consumption. In [38], both CNNs and RNNs were used, whilst in [39], the imaged current’s transient waveform was used as the training set for CNN. Application of long short-term memory (LSTM) NN is present, as well. In [40], the authors proposed adaptive bidirectional LSTM models and showed their superiority against other models, whilst in [40], parallel LSTM topology was proposed. In [41], authors pointed out the potential that deep learning has shown in NILM, solving the problem of vanishing gradient and model degradation using dilated residual attention network. Furthermore, authors applied a combination of bidirectional temporal convolutional networks and achieved improved precision performances in comparison with the previously existing approaches in [36], whilst [42] achieved it by utilizing an attention-based deep NN. Moreover, in [43], a hybrid solution consisting of an adaptive thresholding event detection method, CNN, and kNN, was proposed and was envisioned for detecting any number of appliances in real time. Most importantly, this can be efficiently run on the edge. Taking the presented conclusions from these authors into consideration, it was decided to use a specific deep CNN architecture in this paper, trained by a predefined process using labeled and unlabeled data, to synthesize the beneficial characteristics of both a supervised and unsupervised approach, with the hopes of improving the generalization performances, which turned out to be achievable as the results will show.

In [44], the sequence-to-point (seq2point) CNN architecture was presented. The main idea was to estimate the power consumption of an individual appliance according to the input window of aggregated power so that the output of the network predicts the appliance’s power usage at the midpoint of the window. As this approach showed high performances when compared to previous work, it was selected as the base for work in this paper. Namely, the goal was not to estimate the device’s consumption but to detect its (in)activity. Since this architecture was capable of extracting features necessary for resolving the regression problem, it was expected to perform even better in the context of binary classification. Therefore, in this paper, it was combined with the domain adversarial neural network approach (DANN) [45] to improve generalization performances, with details presented in Section 4.

## 3. Problem Definition

In general, it is widely accepted that good generalization is one of the crucial characteristics that the proposed NILM algorithm should have and is highlighted as one of the five main challenges in [46]. Due to that fact, most of the research tested the proposed solutions on data from houses that were not seen during the training process. However, those houses are, almost always, from the same dataset as the training ones. Therefore, the correlation between the training and testing data is high. More specifically, publicly available datasets consist of measurements from neighboring houses, which are likely to use similar devices, have similar habits, etc. So, even though the testing process is not performed on the house that the algorithm was trained on, the obtained results do not depict the real picture of real-world applications. In other words, since the algorithm is meant to be implemented in practice on houses that do not have smart meters on every appliance, the obtained results would not be adequate and representative and it is highly likely that the expected performances would decrease significantly. Exactly this was highlighted as the key remaining problem to be explored for NILM solutions by [47] review paper. Solving this exact problem is the main focus of this paper. Namely, the proposed algorithm presents an advancement of the seq2point architecture and is supposed to improve generalization on unexplored houses. Moreover, to exploit the full potential of available information, unlabeled data that are usually unused was included, which is one more improvement that this paper contributes to the state-of-the-art algorithms.

In this paper, the underlying idea is the usage of the domain adversarial neural network approach (DANN) to overcome the problem of poor generalization, keeping in mind the fact that there are substantial differences between training and testing data. DANN was inspired by the generative adversarial network (GAN) [48] and was proposed in [45]. The GAN is a widely accepted technique used for generating new data samples, especially popular in the field of image processing, such as in [49,50]. However, due to their high performance in image processing, they were also exploited in other domains. For example, [51] utilized the GAN approach for data imputation in the transportation domain, whilst [52] reviewed generative models for the graph generation. This wide acceptance of GAN influenced further improvement in the field, such as with the DANN proposition. What these two approaches have in common is their way of measuring and minimizing the disparity between the distribution of training data and the synthesized or testing batch. In both cases, the goal is to disable a part of the model from determining the originating domain of the data. For DANN, it is crucial because the aim of the proposed method is not to allow the system to specialize in the training data. In other words, this net is intended for problems where:

- There is a **significant disparity in distribution** between the source and target domains.
- When **labeled** data from the source domain **are** accessible.
- When **labeled** data from the target domain **are not** accessible.
- When **unlabeled** data from the target domain **are** accessible.

In those cases, two of DANN’s main advantages are as follows:

o DANN is able to adapt to the target domain with minimal labeled data. This is particularly useful when labeled data are scarce or expensive to obtain in the target domain.
o DANN is able to minimize the domain discrepancy. DANN is able to learn a shared feature representation that is common across different domains, which is useful in minimizing the domain discrepancy and improving the generalization performance of the model.

This is indeed the case in the considered NILM scenario. Exactly that is why this method is more applicable and shows higher scores, as will be proven in this paper. Namely, many state-of-the-art solutions succeed in achieving respectable results on the tested data. Nevertheless, if the data collected for the place of interest are not large enough, the performances would probably be unsatisfactory and incomparable with the presented ones. Therefore, to make NILM more accessible in everyday life, an algorithm that does not require inaccessible labeled data from the target domain, precisely individual appliance consumption from the target domain, is proposed and, at the same time, performances on that very same domain were increased.

As one of the most impactful contributions of this paper, the DANN method was used for the first time as the core method for solving the NILM problem. Moreover, its performance was benchmarked against seq2point in two scenarios with different datasets, thus emphasizing the generalization potential of the proposed methodology. The semi-supervised approach allows this technique to retain the best characteristics of both supervised and unsupervised methodologies, especially having in mind that the full amounts of available data are utilized.

## 4. Proposed Approach

The DANN architecture was presented in [45] and it has already been used for speech recognition as in [53,54], as well as for image classification [45]. However, it has not been used for solving NILM problems yet, which is one of the contributions of this paper. In this section, the DANN architecture and its training process are going to be briefly discussed, as well as its characteristics and performances.

**Figure 1:**
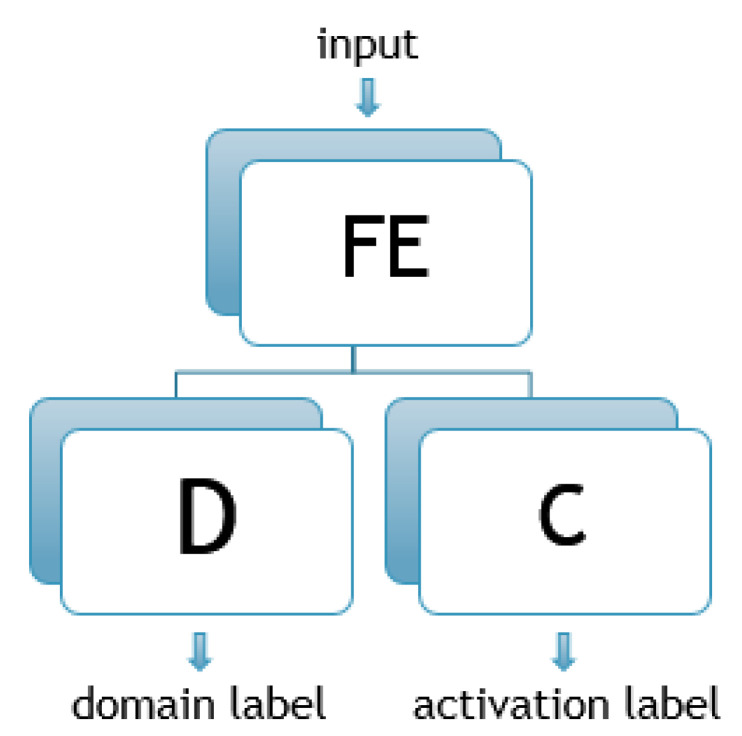
DANN architecture (FE—feature extractor, C—classifier, D—discriminator).

DANN is defined as a neural network consisting of three subnet components—a feature extractor (FE), a classifier (C), and a discriminator (D), as shown in Figure 1. FE is a (C)NN that is supposed to extract features from the input data. In the case of NILM, depending on the input window of aggregated sequence, the outputs of this subnet are different features. If $x=[x_{1},x_{2},...,x_{n}]$ is an input vector representing aggregated power consumption samples, the output of the FE block *f* could be given as

(1) f=F(x).

where F is FE’s mapping function from its inputs to the extracted features given through its adjustable parameters FE. This part of the system is consistent with the previously mentioned seq2point net. The architecture is described in Table 1 with the only difference from seq2point being the omission of the last dense layer as it was used to calculate the appliance’s power consumption according to the extracted features, not for their selection. Hence, the mapping function of seq2point is contained in F, with potentially different values of the parameters due to the different training process. The C is a set of two dense layers, as shown in Table 2, and it is supposed to determine the appliance’s state, given the extracted features by the FE subnet. In other words, the estimated appliance’s state $\dot{s}$ is given as

(2) $$\dot{s}=C\left(f\right).$$

where C is C’s mapping function, represented by parameters *C*. The D is a domain classifier formed alike C, as described in the aforementioned table. D is supposed to distinguish whether the input window of the aggregated sequence originates from the source or the target domain, which is also consistent with the extracted features, which are outputs of the FE. It could also be represented in the following way

(3) $$\dot{d}=D\left(f\right).$$

where $\dot{d}$ corresponds to the estimated origination label and D is D’s mapping function. D is given by parameter *D*.

**Figure 2:**
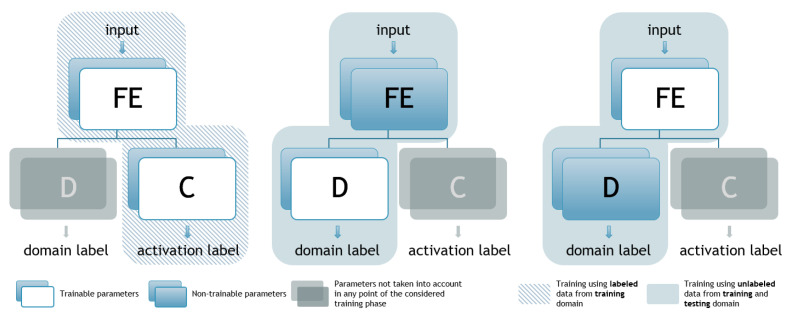
DANN training process (FE—feature extractor, C—classifier, D—discriminator).

The main idea is to direct FE to adjust its parameters during the training process in such a way that the learned features, used by C for activity classification, are not specific to the source domain. This is achieved by training the FE in such a way as to provide outputs that would result in the D part of the net not being able to distinguish correctly the originality of the inputs. Hence, extracted features are supposed to be representative so that C is able to recognize the appliance’s pattern and its activity, but not the source domain, so that D is not able to distinguish the input’s originality at the same time. Selection of the appropriate features for activity classification is achieved using labeled data, whilst preventing specialization on previously seen data and improving generalization is achieved by using unlabeled data for determining the testing set’s probability distribution. In practice, the data are accessible, as each house using any NILM system must provide, at the bare minimum, aggregated power measurements. Hence, not only does this system improve generalization, but it also exploits the full potential of the accessible data. This idea is realized by a specific semi-supervised training process, which is shown in Figure 2 and given by pseudocode in Algorithm 1. It consists of two phases and three steps where, in each one of the steps, two out of three subnets are being trained, whilst the third one is omitted. The steps are given as follows:

1. The FE+C part of the net is trained using a standard backpropagation algorithm, while D is not considered. The aggregated power sequence is taken as an input to the FE block while the output is a hot-encoded vector, referred to as the label, denoting the appliance’s state—active or inactive (10 for active and 01 for inactive), depending on the individual consumption measurements for the considered appliance. In this phase of the training process, only data originating from the source domain are being used, meaning that only labeled data are used. This process is identical to the seq2point training, with all of the same data used. It could be formally given as follows:
  (4) $$FE,C=\underset{FE,C}{\mathrm{argmin}}J\left(s,\dot{s},FE,C\right),$$
  (5) $$\dot{s}=C\left(F\left(x\right)\right),$$
  (6) $$\left(x,s\right)\in S_{labeled}.$$
  where FE is the parameter of the FE network characterizing function F, *C* is the parameter of the C network characterizing function C, *J* is a function used during backpropagation, *x* is the input-aggregated power sequence, *s* is the desired appliance activity label, $\dot{s}$ is the activity estimation, and $S_{labeled}$ represents part of the source domain used for training as labeled data.
2. The second phase consists of two steps in which the FE+D part of the net is trained, whilst C is not considered. The input remains in the aggregated power sequence, whilst for FE+D, the training process outputs are domain-originated hot encoded vectors, or domain labels for short, denoting whether the input sequence comes from the source or target domain. In other words, in this phase of training, no individual appliance consumption is required, or more precisely, no data labels are necessary, implying that this phase of the training process could be considered unsupervised in the context of activity classification. The only data needed are aggregated power measurements.
  (2.1) In the first step of FE+D training, the FE parameters are fixed (non-trainable). In this way, the discriminator’s parameters are adapting to distinguish the domain of origin depending on the features extracted by the FE. The mathematical formalization is as follows:
    (7) $$D=\underset{D}{\mathrm{argmin}}J\left(d,\dot{d},FE,D\right),$$
    (8) $$\dot{d}=D\left(F\left(x\right)\right),$$
    (9) (x,d)∈SD.
    where *J* is the function used during backpropagation, SD represents a dataset containing aggregated power sequences *x* from both the source and target domains and their corresponding domain labels *d*, $\dot{d}$ is the estimated domain label, FE is the parameter of the FE network characterizing function F, and *D* is the parameter of the D network characterizing function D.
  (2.2) After D’s parameters are adjusted, in the last step, they are left to be non-trainable, whilst only FE ones are trained. In this part of the training process, the reversal layer (RL) is supposed to be added between FE and D in accordance with [45]. The motivation for this modification is the fact that the extracted features should not be dependent on the domain, so the discriminator is not supposed to be able to classify them correctly. However, to be able to employ the standard (C)NN training procedure, instead of introducing the RL, in the third phase, all of the domain labels were inverted, resulting in the same effect as the RL would give. Mathematical formalization is as follows:
    (10) $$FE=\underset{FE}{\mathrm{argmin}}J\left(d^{\prime },\dot{d},FE,D\right),$$
    (11) $$\dot{d}=D\left(F\left(x\right)\right),$$
    (12) (x,d)∈SD,
    (13) $$d^{\prime }=d^{-1}.$$
    where *J* is function used during backpropagation, SD represents a dataset containing aggregated power sequences *x* from both the source and target domains; their corresponding domain labels *d*, $d^{\prime }$ are the inverted domain labels to label *d*, $\dot{d}$ is the estimated domain label, FE is the parameter of the FE network characterizing function F, and *D* is the parameter of the D network characterizing function D.

Finally, the sequences of these two phases are repeated until convergence. Each phase/step might be repeated a couple of times within one cycle with the details on the number of epochs and repetitions of each phase in the employed training process given in Section 6.

**Figure 3:**
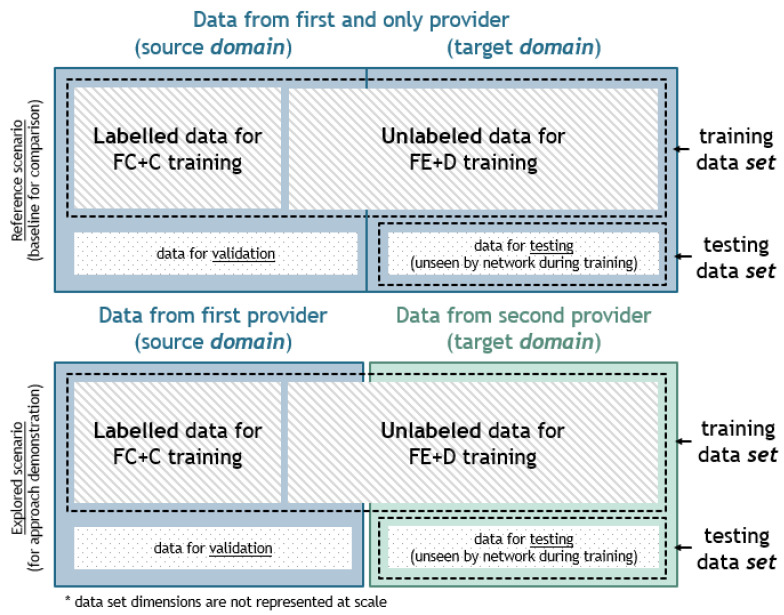
Domain vs. set.

A crucial thing to note is that the terms *set* and *domain* are used to refer to different types of data classification, as shown in Figure 3. Namely, training and testing sets are terms used specifically for the group of data used for either training or testing purposes. In other words, the training *set* is equivalent to data seen by the NN during training used for the NN parameter adjustment and the testing *set* is equivalent to data unseen before being used only for the evaluation of NN’s performances. On the other hand, source and target *domains* refer to the origination of the data where the source domain is the main source of the data and the targeted domain is a dataset with a significantly different distribution than the training one and to which the methodology is to be applied. As already mentioned, labeled data used for the training process come only from the source *domain*. For example, data from one provider can be considered the source domain as it is the main source of the data, used both as labeled and unlabeled, and data from the second provider can be considered the target domain, used only as unlabeled. However, to overcome the issues that stem from the distribution variety, the proposed approach uses data from both source and target *domains* for training purposes. Therefore, the training *set* consists of labeled data from the source *domain* but also incorporates a portion of unlabeled data from both the source and target *domain*. However, when testing performances are presented at the end of this paper, only previously unseen data (testing *set*) were used for the given analyses.
**Algorithm 1** DANN Training    **Inputs:**initial source domain S, target domain Thyperparameters for FE, C, and D networks $h_{FE},h_{C},h_{D}$number of epochs for three steps of the training process $n_{1},n_{2},n_{3}$number of training cycles *n*    **Algorithm:**    $S_{labeled},S_{unlabeled}\leftarrow$ separate data from S for labeled and unlabeled training    $X_{FEC}\leftarrow$ extract inputs from $S_{labeled}$    $Y_{FEC}\leftarrow$ extract outputs from $S_{labeled}$    $X_{FED}\leftarrow$ combine inputs from T and $S_{unlabeled}$    $Y_{FED}\leftarrow$ create domain labels from T and $S_{unlabeled}$    FE,C,D← initialize networks with $h_{FE},h_{C},h_{D}$    **for** i < n **do**    FEC←FE+C    train FEC by $\left(X_{FEC},Y_{FEC}\right)$ for $n_{1}$ epochs    FE← FE part of FEC    C← C part of FEC    FED←FE+D    train FED by $(X_{FED},Y_{FED}$ for $n_{2}$ epochs with FE part frozen    D← D part of FED    $Y_{FED_{inv}}\leftarrow$ invert $Y_{FED}$    train FED by $(X_{FED},Y_{FED_{inv}}$ for $n_{3}$ epochs with *D* part frozen    FE← FE part of FED    **end for**

Taking all previous into consideration, as far as the NILM problem is considered, the authors find this approach adequate according to the fact that it is highly likely to have differently distributed source and target domains, which might lead to significantly decreased performances when using standard approaches. Moreover, it is crucial that DANN is not more computationally complex than the state-of-the-art solutions, as will be presented in Section 7. Therefore, it can be implemented on real data.

## 5. Data Preprocessing and Analysis

In this section, the considered devices, preprocessing methods, and dataset analysis will be presented and discussed.

### 5.1. Input Data Preprocessing

**Figure 4:**
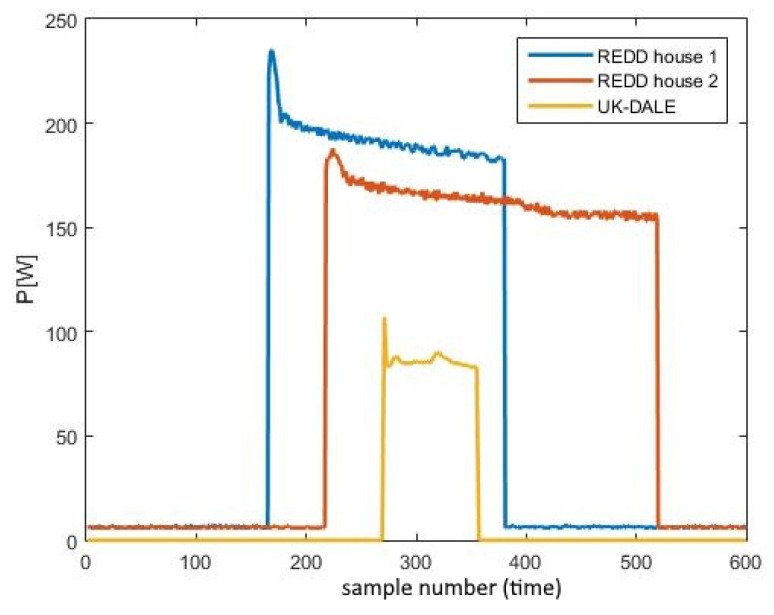
Comparison between the refrigerator’s individual consumption measurements from the REDD and UK-DALE dataset.

Firstly, the considered devices will be analyzed. To be able to implement the proposed method, data from two different domains were required, leading to the necessity of obtaining two different datasets. It was essential to source differently distributed data to be able to test and compare the generalization potential of the proposed architecture. The first dataset was selected to be the REDD dataset [55] intended for the source domain, obtained in the USA, whilst the second was the UK-DALE dataset [56] intended for the target domain, obtained in the United Kingdom. Being geographically far apart, it is expected that both habits and types of domestic appliances significantly differ, leaving enough room to prove the generalization ability. Apart from expecting them to deviate, these datasets are the ones most commonly used among NILM researchers. The examples of the refrigerator’s individual consumption measurements from these two datasets are shown in Figure 4. It can be seen that there is a slight difference in amplitude and activity duration even between the appliances originating from the same dataset. However, the difference between the consumption from the UK-DALE fridge and the REDD ones is much higher both in duration and amplitude. Nonetheless, the decreasing exponential shape as the characteristic signature of the fridge’s consumption persists, therefore allowing that system to recognize them and adequately classify the appliance, but leaving room to demonstrate the generalization potential.

**Figure 5:**
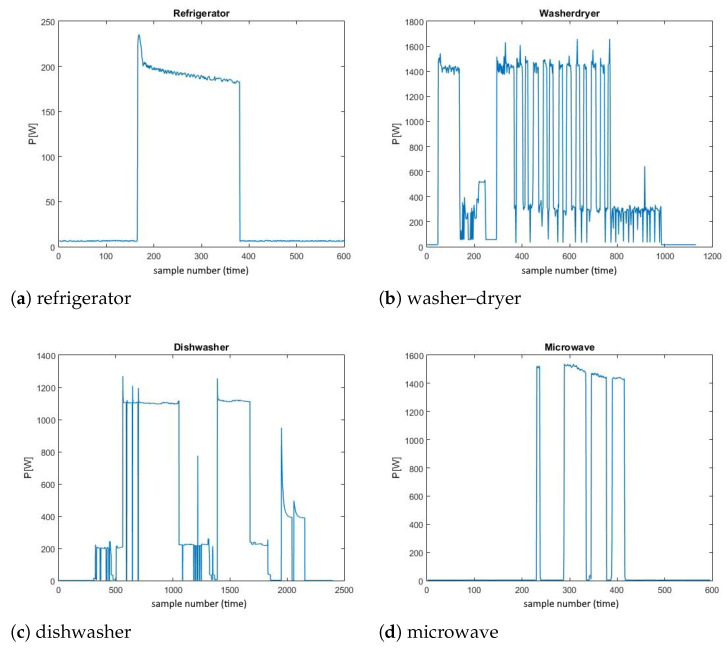
Examples of individual consumption measurements of the appliances.

Taking into consideration the selected datasets and the proposed training process, it was clear that only the devices that are available in both datasets were to be considered. The ones that are included in both of them are refrigerator, washer–dryer, dishwasher and microwave. In order to include as many testing scenarios as possible, all four of them were considered in this paper, and examples of windows of their individual power consumption measurements from the REDD dataset are given in Figure 5.

**Figure 6:**
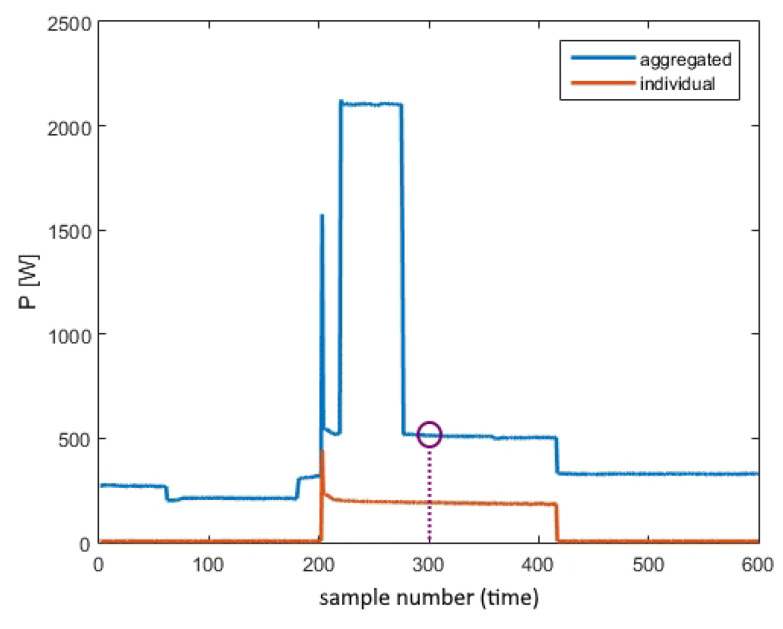
Example of label-defining for the refrigerator.

The used preprocessing algorithms can be classified as standard when neural network training is concerned. Firstly, it was necessary to match samples of aggregated and individual consumption. Namely, in the REDD dataset, the base sampling period for aggregated power was one second, whilst it was three seconds for individual consumption. Moreover, there were time stamps for both databases in which either total or individual power consumption measurements were missing, so it was necessary to retain only the samples that correspond to time stamps in which both measurements were accessible. Secondly, UK-DALE data needed to be up-sampled to obtain equal sampling periods in both domains. Eventually, the input data were selected to be a windowed sequence of M=599 samples, consistent with [44] for all appliances. Depending on the practical application, this can be changed in accordance with the expected appliance’s activity duration and it should not influence the main goal of this paper. Following this, the input data were normalized and centered on zero to bring the inputs close to values that would not lead to divergence or cause the gradient vanishing problem while training. Because the examples of specific appliance inactivity are more common than activity, and to provide a balanced training set, the number of input training data windows was determined depending on the number of appliance’s activations. There are an equal number of examples of active and inactive instances. Finally, the labels were defined depending on the value of the device’s individual power consumption and the predefined threshold shown in Table 3. The described classification process is depicted in Figure 6, showing that the system is supposed to determine whether the considering device is active or inactive in the middle of the aggregated sequence window depending on the aggregated measurement window. It is important to point out that the system has a decision-making delay of 15 minutes, the same as in [44]. Namely, if an appliance’s activation is to be estimated at a point in time denoted *t*, shown in the figure as a circle, it is necessary to obtain the aggregated power consumption for a time interval from $t-T_{w}/2$ to $t+T_{w}/2$ as an input sequence, where $T_{w}$ is the time duration of the input sequence. The idea is to recognize typical appliance patterns which are only possible if such patterns are included in the input sequence, i.e., the decision-making delay is large enough for the pattern to show within the given window. However, considering real-world NILM applications, it can be concluded that this delay does not affect the usability of NILM models and their exploitation by the end users.

### 5.2. Dataset Analysis

Before starting the training process, it was essential to analyze the available data. Therefore, in this subsection, some fundamental characteristics that influenced the results presented in Section 7 are introduced and discussed.

**Figure 7:**
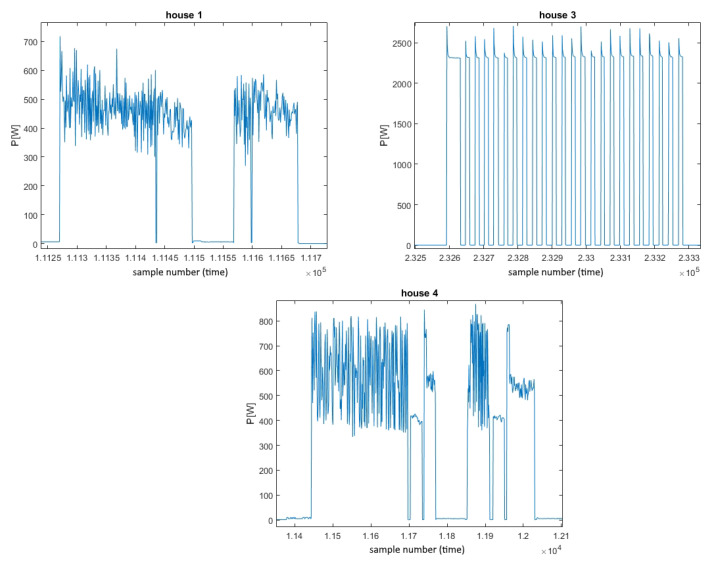
Washer dryer power consumption from different REDD dataset houses.

First, even though for most of the data examples correlation between individual consumption measurements from different appliances of the same type within one dataset are quite high (as shown in Figure 4), when analyzing washer dryer measurements from different houses within the REDD dataset, notable diversity can be observed in both amplitude and shape, as shown in Figure 7. As is obvious here, data from the third house are not correlated at all with the others. As clearly shown here, measurements from the third house do not have the same specific patterns as the others, indicating possibly a completely different type of appliance. Namely, in connection with the previously discussed disparity between the datasets, it was highlighted that despite the difference in amplitudes, durations, presence of noise, etc., characteristic patterns should be visible, which is not the case here. Therefore, it might even be the case that measurements from the third house are badly labeled and that they do not correspond to a washer dryer. Hence, the data from house 3 were considered as outliers and were omitted before the training process. Finally, all other available REDD data were used in experiments, either for training or testing purposes.

Second, when considering correlation, a fundamental aspect of this paper, and its impact on improving generalization, the microwave has clearly distinguished itself. Namely, the lack of differences between the domains for this device result in an unexpectedly high correlation. To determine how strongly the correlated microwave power consumption from different datasets was, a couple of different metrics and methods were employed. Power consumption signals, more precisely their similarities and differences, were first analyzed and the results are going to be introduced. Mean stationary consumption for REDD and UK-DALE microwaves were compared. Namely, the stationary value of REDD’s microwave is approximately 1550 W, whilst UK-DALE’s microwave is around 1600 W, leading to the conclusion that the relative difference is roughly 3% of the mean stationary value, which can be regarded as insignificant.

**Figure 8:**
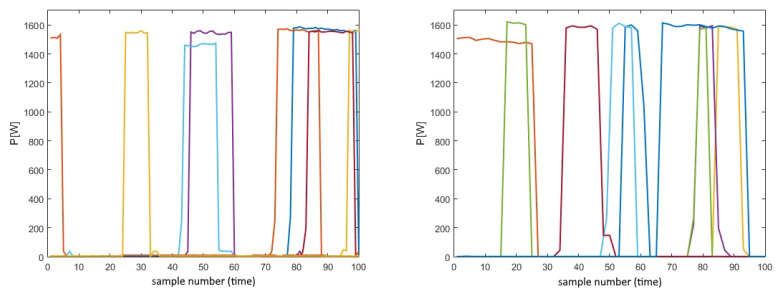
REDD’s (1) and UK-DALE’s (2) examples of microwave power consumption.

Apart from proving that disparity in the stationary level of power consumption practically does not exist, it was necessary to analyze consumption patterns, as the only remaining characteristics that can vary between these two domains. More than a thousand widow sequences of microwaves’ power consumption from both datasets were analyzed, and it was concluded that in both cases the microwaves’ power consumption signature can be well approximated with the rectangular pulse. Therefore, given that the heights of these pulses are almost the same, having in mind the level of power consumption in the steady state, the only arguable parameter left is their duration. Taking into consideration that the microwave can be used for a wide range of purposes, from warming up the bread, which takes only a couple of seconds, to defrosting, which takes around half an hour, it is expected that the duration in both sets varies a lot. By closely analyzing the data, it was derived that in the REDD dataset duration varies from 15 s to more than 30 min, which has led to the conclusion that the net has seen a wide range of possible inputs, so it should perform well no matter how long or short the testing duration is. After this, UK-DALE data were analyzed for the duration as well. It can be said that there are slightly more examples of short activity durations than in the REDD set. However, all durations are consistent with the ones from the REDD set, leading to the conclusion that the target domain is highly similar to the training one, implying that high correlation scores are expected. All previously mentioned characteristics can be seen in Figure 8, which presents a couple of the analyzed examples, as it is not easy to transparently and systematically show all of them in one picture. Namely, it is clear from them that determining the originating domain is very difficult, leading to the conclusion that they are highly correlated.

Nonetheless, to corroborate the assumptions from the given visual analysis, it was necessary to accompany these correlation descriptions with some widely accepted metrics. The first metrics used were the Pearson cross-correlation coefficient between consumption, calculated as

(14) $$\rho =\frac{\sigma_{xy}}{\sqrt{\sigma_{xx}\sigma_{yy}}}$$

where

(15) $$\sigma_{xy}=\frac{1}{N-1}\sum_{i=1}^{N}\left(x_{i}-\bar{x}\right)\left(y_{i}-\bar{y}\right)$$

where *N* is the number of samples in sequences *x* and *y*, and $\bar{x}$ and $\bar{y}$ are their mean values. The estimation was computed as the mean value of the maximum correlation coefficient between the window of individual power consumption of the microwave from the UK-DALE and all of the windows of REDD’s microwave. After calculating this correlation for each house from the REDD dataset, the obtained results were averaged. The final estimated value of the correlation coefficient turned out to be 0.8212, which was accepted as very high.

Moreover, similarities in the spectral domain have also been analyzed. For every previously mentioned window, Fourier’s transformation was calculated. The selected metric was defined as the mean value of the minimum number of square errors between the amplitude spectrum of one UK-DALE window spectrum and all of the REDD window spectrums for each UK-DALE window. To facilitate the validity of the comparison between different appliances, the data were first normalized by the mean of the individual devices’ consumption from the training set. As there is no widely accepted threshold for this metric to determine whether signals are correlated, this analysis was conducted for all four devices, and the results are shown in Table 4. It can be observed that the minimum mean square error (MSE) corresponds to the microwave, implying the fact that their spectrums are the most similar, concluding that in the case of the microwave, the used two domains do not have different enough distributions, implying that the generalization potential for this appliance is very low.

Moreover, the last compared metric (that also supports these conclusions) was the mean energy of the windowed signal. In Table 5, the ratio $a_{1}$ between average energy per window of REDD device $E_{\mathrm{REDD}}$ and the average energy per window of the UK-DALE device $E_{\mathrm{UKDALE}}$ and corresponding reciprocate value $a_{2}$ are given. It is clear that the smallest deviation is for the microwave, leading to one more justification for the claim that the microwave’s signals are highly correlated.

## 6. Model Training and Implementation

To completely describe the training process, important training parameters are given in Table 6. Net’s training was implemented in Python using the TensorFlow library which enables the parallelization of the training process on the GPU (NVIDIA GeForce 1080 Ti), which significantly accelerated it. Two different architectures were trained (seq2point and DANN) and training analytics are going to be presented and deliberated on in this section.

To provide a comparison between the state-of-the-art solution (seq2point) and the proposed DANN approach with improved generalization, the data were organized as follows in one of two ways for all four appliances (Figure 3):

1. Only REDD data were used. More precisely, a part of it was used as the source and a different part of it (data from one house) as the target *domain*.
2. Both REDD and UK-DALE data were used with REDD as the source *domain* and UK-DALE as the target *domain*.

Seq2point was trained on a training *set* using only the portion of the data from the source *domain*, which came from REDD. The performances of this network were used to provide a baseline for comparison with DANN, which was trained afterward. On the other hand, DANN was trained using data both from the source and target *domains*, as explained in Section 4. Testing of the two different cases was implemented to show the potential of the proposed approach when compared with a case where the generalization potential is low. Therefore, in the first case, generalization potential was low since the data from the source and target domains had a similar distribution, as both originated from the same dataset. On the other hand, in the second scenario, the generalization potential was higher, as data from the source and target domains had different distributions, which was utilized to show how the model would perform in a real-world setting.

Nevertheless, apart from the fact that, in these scenarios, training was performed using different data, this process can be considered as basically the same. In the manner that was presented in Section 4 in Figure 2, training for this architecture consists of 2 phases—one that corresponds to the classifier training (left diagram) and the other that is associated with the discriminator fitting (middle and right diagram). However, the ratio between classifier and discriminator training length can be extremely important. In other words, if the classifier is not trained long enough, it cannot extract any meaningful features from either source or target domain, resulting in underperformance. Conversely, if insisting on classifier training, it will end up as if the discriminator had not existed. Taking all of the previous into consideration, it can be concluded that it is relevant to train the classifier for some time longer than the discriminator as its task can be considered more challenging. Therefore, in this paper, it was experimentally concluded that in each cycle it was optimal to carry out phase one of training two times in a row, whilst the second was performed only once every cycle. In the final epoch, only phase one was carried out.

Finally, the training process was conducted and training terminations for all 12 of the considered nets are elaborated in Table 7. Starting with the seq2point refrigerator’s architecture, the training process was stopped as the criterion value started increasing and this metric, which previously reached almost 98% on both the training and validation set, started decreasing. Namely, this occurs when the algorithm has reached a small region around the optimal solution and it cannot reduce the function any further. The very same idea was applied to the DANN trained for classifying dishwasher’s activity when trained on the REDD data. Considering the DANN trained for refrigerator’s activity classification on REDD data, termination occurred as both the training and validation criteria did not change significantly for several epochs. The same was for the washer dryer’s seq2point and DANN on REDD, and for all microwave’s nets. Furthermore, the last net trained for the refrigerator’s activity detection, the DANN using both REDD and UK-DALE sets, was terminated due to the fact that the validation metric did not change for a couple of epochs, whilst it increased on the training data, leading to an increasing difference between the training and validation criteria, which indicates overfitting. Moreover, the washer dryer and dishwasher’s DANN (trained on combined data) were stopped because of fact that the validation criterion increased. Finally, the dishwasher’s seq2point training process was stopped due to the fact that an already significant difference between training and validation criteria was further increased by increasing validation criteria for a couple of epochs.

## 7. Performance Comparison and Results

After defining the datasets that were used, architectures and training procedures, preprocessing algorithms, and analyzing the data for generalization applicability, this section provides the results of the proposed methodology and discusses them in detail. In order to appropriately compare the performances of seq2point and DANN architectures, two scenarios were tested. Namely, the main idea for DANN implementation was improving generalization. Therefore, the essential disparity between the two proposed scenarios was the difference between the source and target domains.

In the first one, both source and target domains were subsets of the REDD dataset, as presented in the above picture in Figure 3. For the target domain, data from one house for each appliance were separated, so that the obtained testing performances would truthfully depict the system behaviors on the unseen house, while the remaining houses formed the source domain. Hence, in this first scenario, data from the separated house were partially used for the training set, but only as unlabeled, while the rest were used for the performance evaluation during the testing phase (testing set). Finally, such a partition resulted in the fact that none of the data used for the training process were used for testing. As for the second scenario, the REDD set represented the source domain and UK-DALE represented the target domain. This resulted in significantly different data distributions between the domains and enabled higher generalization potential. Similarly to the first scenario, as given in the picture below in Figure 3, the target domain was split, so part of it was used as unlabeled during the training process, and part of it was used only for the performance evaluation.

House combinations used for source and target domain purposes are shown in Table 8. The initial idea was to use the same house for all testing purposes. However, this was not possible for several reasons. Considering the REDD as the target domain, data from the third house for washer–dryer were not correlated enough with all the others, as discussed in Section 5. As these data were not taken into account any further, the washer dryer’s REDD testing house was selected differently from the others. It should be mentioned that the only house that contained data from all of the analyzed appliances was the first one. Nonetheless, for all of the devices, the most comprehensive data originated from this house, which is why it was saved for training purposes. Furthermore, considering the UK-DALE dataset, it was not possible to use the same house, as none of the available houses contained measurements of all of the considered appliances. Therefore, for the washer–dryer the only possible (fifth) one was selected, whilst for all of the others testing data originated from the same (first) UK-DALE house.

Finally, the performance comparison is presented in Table 9. There, one could observe F1 scores, depicting how models are accurate, both for the seq2point and DANN architectures for all four scenarios per each device. In the first scenario, the seq2point trained networks were tested on unseen REDD houses, and in the second scenario, they were tested on selected UK-DALE houses. Except for the microwave, the seq2point’s performance significantly declined when tested on different domains, by more than 25% in some cases. As previously mentioned, DANN was also tested in these two scenarios. In the first one, since the data from the source and target domain were expected to be highly correlated, it was expected that the seq2point network would outperform DANN for all devices, which was confirmed. This is because CNNs perform better when the training set represents the problem well, meaning that all relevant data are included. In cases where there is little difference between the training and testing data, CNNs are a suitable solution. On the other hand, DANN is inadequate in this context because it is meant to be applied to problems where significant differences in domains exist. In this case, the lack of differences leads to a decrease in the information used for classification without a valid reason, resulting in inferior performance.

On the other hand, when comparing F1 scores on the testing data set coming from differently distributed domains in the feature spaces, it is expected that DANN outperforms seq2point. This was corroborated by the second tested scenario, where for 3 out 4 available devices, DANN outperformed the seq2point architecture for 2% on average, proving its crucial generalization capability. These results are consistent with other previously published results from the DANN applications in other domains. Similar to these results, in [53], the authors achieved an error rate reduction in speech recognition of around 2%. The fact that DANN outperformed seq2point, in this case, was the consequence of the fact that DANN’s architecture, and the corresponding training process, contributed to more adequately choosing features and, thus, improving accuracy performances. However, when testing on the microwave, seq2point performed better because the power consumption of microwaves was found to be highly correlated between the two domains, as discussed previously. One characteristic that contributes to this assertion is the fact that the seq2point architecture trained on REDD houses achieved better performances on the UK-DALE house than on the REDD unseen one (as shown by seq2point’s 82% and 89% F1 scores for the microwave). Therefore, it is expected that UK-DALE is more similar to the training data, implying a high correlation between the two domains. Moreover, as proven in Section 5.2, there is not much of a difference between the REDD and UK-DALE microwave’s data, suggesting that the most important premise on differently distributed domains was not satisfied for this appliance. Despite this exception, the study suggests that this approach is improving current state-of-the-art performances and has the potential for practical use in improving generalization performance across various devices.

After proving that the DANN approach can improve generalization performances, it was necessary to show that the training DANN duration is comparable with the seq2point one, so that this improvement in generalization does not come with a drastic cost in the training time. Keeping in mind that, for each appliance, a different number of training examples was present, it was decided to compare normalized training duration instead of the absolute one. Additionally, keeping in mind that the FE+C and FE+D parts of the DANN architecture were trained using different data, resulting in an unequal number of training examples, the final estimation of metrics is given as

(16) $$m_{\mathrm{seq}}=t_{\mathrm{seq}}/k_{\mathrm{train}},$$

(17) $$m_{\mathrm{DANN}}=t_{\mathrm{FE}+C}/k_{\mathrm{train}}+t_{\mathrm{FE}+D}/k_{\mathrm{test}},$$

where $m_{x}$ represents the metrics for the x architecture, $t_{x}$ time spent training x architecture, $k_{\mathrm{train}}$ number of examples from the source domain used for seq2point and FE+C net training and $k_{\mathrm{test}}$ number of examples from the target domain used for the FE+D training process. Finally, the ratios between $m_{\mathrm{seq}}$ and $m_{\mathrm{DANN}}$ are shown in Table 10 as the relevant metrics for the considered household appliances. Here, it can be observed that, on average, 2.44 times more time has to be invested into the training process in the case of DANN in comparison with the seq2point architecture. However, this is completely acceptable having in mind that this is a one-time effort that can be handled within one day.

Finally, as the last step of the performance evaluation that will be given in this paper, analysis related to the ability of this solution to run, not only on the cloud, but on the edge. This is crucial to confirm the potential for large-scale rollout of this approach. The most important fact related to applying DANN on the edge is that it does not introduce any additional restrictions to conventional deep NN models edge application, since the difference which improves the generalization performance is related to the training, not the running process. During the running phase, it is a standard NN model, which calculates the outputs of each hidden layer, and finally, it combines it into the output. The restrictions that should be considered, though, as for any NN edge application, are related to the memory limit, especially when architecture is growing. Hence, it might be the case that some complex NN architectures would have to be omitted when applied to some simple edge computer, even though they could be easily run on the cloud. However, this would be a limitation even if the same sequence-to-point architecture is applied. Hence, it could be concluded that DANN is applicable on both the edge and cloud platforms, respecting available resources in the same manner as any NN.

Taking all of the previous into consideration, it can be concluded that this novel DANN approach in the NILM field can result in a noticeable improvement in performance in a real-world practice with acceptable costs in terms of training and execution times.

## 8. Conclusions

Convolutional neural networks are widely used for numerous classification and regression problems as they achieve high performance, even when problems are convoluted. Nonetheless, their performances are highly dependent on the data used, particularly if the testing data deviate significantly from the training set when performance can significantly decrease.

Considering the NILM problem, publicly available datasets can deviate from the measurements of interest depending on the location, the number of household members, social status, etc. Therefore, CNN’s performance on the house of interest tends to be unacceptably low because of the fact that the training set does not represent the target domain adequately. On account of this problem, this paper presents a method that improves generalization capabilities by additionally using unlabeled data from the target domain, which are always available and often left unused. In particular, domain adversarial neural networks were implemented for the first time for solving the NILM problem and improving the disaggregation generalization performance, by several percent. Namely, the generalization potential was verified using the two most frequently used datasets: REDD and UK-DALE, concluding that this method improves current state-of-the-art solutions and can be suggested for practical use. Moreover, if this approach is to be used in a real-world application, it could potentially achieve even higher precision, since models could be retrained using unlabeled data from the target domain, which are constantly growing.

Even though the main goal of proving that the proposed approach improves the generalization performance was achieved within this paper, additional research potential is still present. Namely, it was stated that the approach presented within this paper does not provide real-time energy disaggregation, which is one of the downsides that could be improved and analyzed in the following research. Moreover, the authors firmly believe that semi-supervised approaches, although highly suitable in the given context, are still underrepresented in literature and, therefore, present a potential area of future research. Furthermore, since NILM is a hot topic nowadays, many open fields of research have not been tackled in detail within this paper. The robustness of the algorithms in the presence of appliances with similar consumption patterns should be analyzed since it could affect the performances in real-world applications. Moreover, improvements are desired in providing affordable smart meters with high sampling rates, to create many opportunities for improving disaggregation algorithms with techniques that cannot be used with presently available measurements.

## Figures and Tables

**Table 1:** Feature extractor’s layers.

Layer	No. Ker.	Ker. Size	Activation	Ratio
Conv1D	30	10	ReLU	
Dropout				0.5
Conv1D	30	8	ReLU	
Conv1D	40	6	ReLU	
Dropout				0.5
Conv1D	50	5	ReLU	
Conv1D	50	5	ReLU	
Dropout				0.5
Conv1D	1024	570	ReLU	
Dropout				0.5
Dense	100		linear	

**Table 2:** Classifier and discriminator’s architecture.

Layer	No. of Neurons	Activation
Dense	100	linear
Dense	2	sigmoid

**Table 3:** Thresholds for estimating appliance activity labels.

	Threshold [W]
**refrigerator**	30
**washer dryer**	50
**dishwasher**	30
**microwave**	30

**Table 4:** Comparison of MSE in the amplitude spectrum.

	avg MSE
**refrigerator**	1.2563
**washer dryer**	6.4950
**dishwasher**	1.0201
**microwave**	**0.8053**

**Table 5:** Comparison between energies of individual consumption signals.

	a1=EREDDEUKDALE	a2=EUKDALEEREDD	$\max(a_{1},a_{2})$
**refrigerator**	**3.66**	0.27	3.66
**washer dryer**	**5.18**	0.19	5.18
**dishwasher**	0.41	**2.41**	2.41
**microwave**	**1.80**	0.55	**1.80**

**Table 6:** Training characteristics.

**number of samples in window**	599
**window duration**	30 min
**sampling period of input sequence**	3 s
**decision-making delay**	15 min
**criterion**	MSE
**optimization algorithm**	ADAM
**learning rate**	$10^{-5}$
$\beta_{1}$	0.9
$\beta_{2}$	0.999

**Table 7:** Explanations for training termination.

	seq2point	DANN only on REDD	DANN on REDD + UK-DALE
**refrigerator**	decreasing metrics after reaching almost maximum value	neither training nor validation criteria have changed significantly for several epochs	validation has not changed, training metric decreased, their difference increased
**washer dryer**	neither training nor validation criteria have changed significantly for several epochs	neither training nor validation criteria have changed significantly for several epochs	validation criterion increased
**dishwasher**	an already significant difference increased by increasing validation criterion	decreasing metrics after reaching almost maximum value	validation criterion increased
**microwave**	neither training nor validation criteria have changed significantly for several epochs	neither training nor validation criteria have changed significantly for several epochs	neither training nor validation criteria have changed significantly for several epochs

**Table 8:** House to the originating domain per appliance mapping.

	REDD Train	REDD Test	UK-DALE Test
**refrigerator**	1, 2, 5, 6	3	1
**washer–dryer**	1	4	5
**dishwasher**	1, 2, 4, 5	3	1
**microwave**	1, 2, 5	3	1

**Table 9:** Comparison of the obtained F1 scores.

	only REDD		REDD + UK-DALE	
	**seq2point**	**DANN**	**seq2point**	**DANN**
**refrigerator**	**85%**	71%	59%	**62%**
**washer–dryer**	**85%**	83%	80%	**82%**
**dishwasher**	**88%**	75%	78%	**79%**
**microwave**	**82%**	81%	**89%**	84%

**Table 10:** Ratio of DANN and seq2point normalized training time given the number of training examples.

	$m_{\mathrm{DANN}}/m_{\mathrm{seq}}$
**refrigerator**	1.82
**washer–dryer**	3.19
**dishwasher**	1.70
**microwave**	3.07
**avg**	**2.44 **

## References

[1] (2013). Sustainable Buildings and Climate Initiative (UNEP-SBCI). energies2050.org.

[2] Carrie Armel K., Gupta A., Shrimali G., Albert A. (2013). Is disaggregation the holy grail of energy efficiency? The case of electricity. Energy Policy.

[3] Tekler Z.D., Low R., Zhou Y., Yuen C., Blessing L., Spanos C. (2020). Near-real-time plug load identification using low-frequency power data in office spaces: Experiments and applications. Appl. Energy.

[4] Ridi A., Gisler C., Hennebert J. A Survey on Intrusive Load Monitoring for Appliance Recognition. Proceedings of the 2014 22nd International Conference on Pattern Recognition.

[5] Franco P., Martínez J.M., Kim Y.C., Ahmed M.A. (2021). IoT Based Approach for Load Monitoring and Activity Recognition in Smart Homes. IEEE Access.

[6] Tekler Z.D., Ono E., Peng Y., Zhan S., Lasternas B., Chong A. (2022). ROBOD, room-level occupancy and building operation dataset. Build. Simul..

[7] Tekler Z.D., Low R., Yuen C., Blessing L. (2022). Plug-Mate: An IoT-based occupancy-driven plug load management system in smart buildings. Build. Environ..

[8] Hart G.W. (1992). Nonintrusive appliance load monitoring. Proc. IEEE.

[9] Klemenjak C., Goldsborough P. (2016). Non-intrusive load monitoring: A review and outlook. arXiv.

[10] Henriet S., Simsekli U., Fuentes B., Richard G. (2018). A Generative Model for Non-Intrusive Load Monitoring in Commercial Buildings. arXiv.

[11] Bonfigli R., Principi E., Fagiani M., Severini M., Squartini S., Piazza F. (2017). Non-intrusive load monitoring by using active and reactive power in additive Factorial Hidden Markov Models. Appl. Energy.

[12] Jia R., Gao Y., Spanos C.J. A fully unsupervised non-intrusive load monitoring framework. Proceedings of the 2015 IEEE International Conference on Smart Grid Communications (SmartGridComm).

[13] Yu S.Z. (2010). Hidden semi-Markov models. Artif. Intell..

[14] Kim H., Marwah M., Arlitt M., Lyon G., Han J. Unsupervised disaggregation of low frequency power measurements. Proceedings of the 2011 SIAM International Conference on Data Mining.

[15] Ma Y.J., Zhai M.Y. (2019). A non-intrusive load decomposition algorithm for residents. Neural Comput. Appl..

[16] Wu Z., Wang C., Peng W., Liu W., Zhang H. (2021). Non-intrusive load monitoring using factorial hidden markov model based on adaptive density peak clustering. Energy Build..

[17] Bonfigli R., Squartini S., Fagiani M., Piazza F. Unsupervised algorithms for non-intrusive load monitoring: An up-to-date overview. Proceedings of the 2015 IEEE 15th International Conference on Environment and Electrical Engineering (EEEIC).

[18] Heracleous P., Angkititrakul P., Kitaoka N., Takeda K. Unsupervised energy disaggregation using conditional random fields. Proceedings of the IEEE PES Innovative Smart Grid Technologies, Europe.

[19] Liao J., Elafoudi G., Stankovic L., Stankovic V. Power disaggregation for low-sampling rate data. Proceedings of the 2nd International Non-intrusive Appliance Load Monitoring Workshop.

[20] Zhou Z., Xiang Y., Xu H., Wang Y., Shi D. (2022). Unsupervised Learning for Non-intrusive Load Monitoring in Smart Grid Based on Spiking Deep Neural Network. J. Mod. Power Syst. Clean Energy.

[21] Parson O., Ghosh S., Weal M., Rogers A. (2014). An unsupervised training method for non-intrusive appliance load monitoring. Artif. Intell..

[22] Iwayemi A., Zhou C. (2015). SARAA: Semi-supervised learning for automated residential appliance annotation. IEEE Trans. Smart Grid.

[23] Barsim K.S., Yang B. Toward a semi-supervised non-intrusive load monitoring system for event-based energy disaggregation. Proceedings of the 2015 IEEE Global Conference on Signal and Information Processing (GlobalSIP).

[24] Stankovic V., Liao J., Stankovic L. A graph-based signal processing approach for low-rate energy disaggregation. Proceedings of the 2014 IEEE Symposium on Computational Intelligence for Engineering Solutions (CIES).

[25] Zhao B., Stankovic L., Stankovic V. (2016). On a training-less solution for non-intrusive appliance load monitoring using graph signal processing. IEEE Access.

[26] Zhao B., Stankovic L., Stankovic V. Blind non-intrusive appliance load monitoring using graph-based signal processing. Proceedings of the 2015 IEEE global conference on signal and information processing (GlobalSIP).

[27] Zhai M.Y. (2020). A new graph learning-based signal processing approach for non-intrusive load disaggregation with active power measurements. Neural Comput. Appl..

[28] Li X., Zhao B., Luan W., Liu B. (2022). An unsupervised load disaggregation approach based on graph signal processing featuring power sequences. Proceedings of the BuildSys’22, 9th ACM International Conference on Systems for Energy-Efficient Buildings, Cities, and Transportation.

[29] Tabatabaei S.M., Dick S., Xu W. (2016). Toward non-intrusive load monitoring via multi-label classification. IEEE Trans. Smart Grid.

[30] Ibrahim W.G.M., Alshareef S., Talwar S. (2016). Smart Multi-Purpose Monitoring System Using Wavelet Design and Machine Learning for Smart Grid Applications. U.S. Patent.

[31] Wang A.L., Chen B.X., Wang C.G., Hua D. (2018). Non-intrusive load monitoring algorithm based on features of V–I trajectory. Electr. Power Syst. Res..

[32] De Baets L., Ruyssinck J., Develder C., Dhaene T., Deschrijver D. (2018). Appliance classification using VI trajectories and convolutional neural networks. Energy Build..

[33] Rahimpour A., Qi H., Fugate D., Kuruganti T. (2017). Non-intrusive energy disaggregation using non-negative matrix factorization with sum-to-k constraint. IEEE Trans. Power Syst..

[34] Dinesh C., Welikala S., Liyanage Y., Ekanayake M.P.B., Godaliyadda R.I., Ekanayake J. (2017). Non-intrusive load monitoring under residential solar power influx. Appl. Energy.

[35] Kelly D. (2016). Disaggregation of Domestic Smart Meter Energy Data. Ph.D. Thesis.

[36] Jia Z., Yang L., Zhang Z., Liu H., Kong F. (2021). Sequence to point learning based on bidirectional dilated residual network for non-intrusive load monitoring. Int. J. Electr. Power Energy Syst..

[37] Kelly J., Knottenbelt W. Neural nilm: Deep neural networks applied to energy disaggregation. Proceedings of the 2nd ACM International Conference on Embedded Systems for Energy-Efficient Built Environments.

[38] Paulo P. (2016). Applications of Deep Learning Techniques on NILM. Ph.D. Dissertation.

[39] Lan Z., Yin B., Wang T., Zuo G. A non-intrusive load identification method based on convolution neural network. Proceedings of the 2017 IEEE Conference on Energy Internet and Energy System Integration (EI2).

[40] Kaselimi M., Doulamis N., Voulodimos A., Protopapadakis E., Doulamis A. (2020). Context Aware Energy Disaggregation Using Adaptive Bidirectional LSTM Models. IEEE Trans. Smart Grid.

[41] Xia M., Liu W., Xu Y., Wang K., Zhang X. (2019). Dilated residual attention network for load disaggregation. Neural Comput. Appl..

[42] Piccialli V., Sudoso A.M. (2021). Improving Non-Intrusive Load Disaggregation through an Attention-Based Deep Neural Network. Energies.

[43] Athanasiadis C.L., Papadopoulos T.A., Doukas D.I. (2021). Real-time non-intrusive load monitoring: A light-weight and scalable approach. Energy Build..

[44] Zhang C., Zhong M., Wang Z., Goddard N., Sutton C. Sequence-to-point learning with neural networks for non-intrusive load monitoring. Proceedings of the Thirty-Second AAAI Conference on Artificial Intelligence.

[45] Ganin Y., Ustinova E., Ajakan H., Germain P., Larochelle H., Laviolette F., Marchand M., Lempitsky V. (2016). Domain-adversarial training of neural networks. J. Mach. Learn. Res..

[46] Kaselimi M., Protopapadakis E., Voulodimos A., Doulamis N., Doulamis A. (2022). Towards Trustworthy Energy Disaggregation: A Review of Challenges, Methods, and Perspectives for Non-Intrusive Load Monitoring. Sensors.

[47] Angelis G.F., Timplalexis C., Krinidis S., Ioannidis D., Tzovaras D. (2022). NILM applications: Literature review of learning approaches, recent developments and challenges. Energy Build..

[48] Goodfellow I., Pouget-Abadie J., Mirza M., Xu B., Warde-Farley D., Ozair S., Courville A., Bengio Y. Generative adversarial nets. Proceedings of the Advances in Neural Information Processing Systems.

[49] Isola P., Zhu J.Y., Zhou T., Efros A.A. Image-to-Image Translation with Conditional Adversarial Networks. Proceedings of the 2017 IEEE Conference on Computer Vision and Pattern Recognition (CVPR).

[50] Pumarola A., Agudo A., Sanfeliu A., Moreno-Noguer F. Unsupervised Person Image Synthesis in Arbitrary Poses. Proceedings of the 2018 IEEE/CVF Conference on Computer Vision and Pattern Recognition.

[51] Low R., Tekler Z.D., Cheah L. (2020). Predicting Commercial Vehicle Parking Duration using Generative Adversarial Multiple Imputation Networks. Transp. Res. Rec..

[52] Guo X., Zhao L. (2022). A Systematic Survey on Deep Generative Models for Graph Generation. IEEE Trans. Pattern Anal. Mach. Intell..

[53] Shinohara Y. Adversarial Multi-Task Learning of Deep Neural Networks for Robust Speech Recognition. Proceedings of the Interspeech.

[54] Mirsamadi S., Hansen J.H.L. (2019). Multi-domain adversarial training of neural network acoustic models for distant speech recognition. Speech Commun..

[55] Kolter J.Z., Johnson M.J. REDD: A public data set for energy disaggregation research. Proceedings of the Workshop on Data Mining Applications in Sustainability (SIGKDD).

[56] Kelly J., Knottenbelt W. (2015). The UK-DALE dataset, domestic appliance-level electricity demand and whole-house demand from five UK homes. Sci. Data.

